# Periodontal treatment does not result in detectable platelet activation in vivo

**DOI:** 10.1007/s00784-019-03049-x

**Published:** 2019-08-29

**Authors:** Markus Laky, Isabella Anscheringer, Lukas Wolschner, Stefan Heber, Hady Haririan, Xiaohui Rausch-Fan, Ivo Volf, Andreas Moritz, Alice Assinger

**Affiliations:** 1 Division of Conservative Dentistry and Periodontology, School of Dentistry, Medical University of Vienna, Vienna, Austria; 2 Department for Vascular Biology and Thrombosis Research, Center for Physiology and Pharmacology, Medical University of Vienna, Schwarzspanierstraße 17, Vienna, 1090 Austria; 3 Institute of Physiology, Center for Physiology and Pharmacology, Medical University of Vienna, Vienna, Austria

**Keywords:** Periodontitis, Periodontal treatment, Platelet activation, Cardiovascular disease

## Abstract

**Objectives:**

Periodontitis is associated with systemic inflammation, elevated platelet activation and enhanced risk for cardiovascular diseases, while periodontal treatment reduces tissue inflammation and shows desirable effects on the oral biofilm and dental health. However, subgingival debridement during conservative treatment can lead to local trauma and transient bacteraemia, which might affect cardiovascular risk in these patients. Therefore, we investigated the effect of periodontal treatment on systemic platelet activation.

**Materials and methods:**

In a prospective therapeutic trial, 26 patients underwent periodontal treatment and patient blood was analysed immediately before and immediately after intervention for platelet activation markers (flow cytometric analysis of P-selectin, CD63 and CD40L surface expression, integrin αIIbβ3 activation and fibrinogen binding, intra-platelet reactive oxygen species production, platelet-leukocyte aggregate formation and intra-platelet vasodilator-stimulated phosphoprotein phosphorylation) in response to adenosine diphosphate (ADP).

**Results:**

The present study shows that basal platelet activation levels remain largely unaltered in response to periodontal treatment. We also did not observe significant changes in platelet reactivity in response to different concentrations of platelet agonist ADP.

**Conclusion:**

Subgingival debridement does not result in relevantly elevated platelet activation. Thus, augmented platelet activation seems unlikely to be a causative triggering factor that increases the short-term risk for platelet-mediated thrombotic events in response to subgingival debridement.

**Clinical relevance:**

Subgingival debridement is a safe procedure and does not increase the short-term risk for platelet-mediated thrombotic events.

## Introduction

Periodontitis, a chronic inflammatory disease of the tooth-surrounding tissues, has been associated with systemic inflammation and elevated platelet activation. Thus, it is recognised as an important underlying cause to boost inflammatory mechanisms which lead to the occurrence of cardiovascular diseases (CVD) [1, 2]. This makes early treatment of periodontitis an important factor to improve oral health.

The basis of periodontal therapy is anti-infective non-surgical treatment with the objective of controlling the oral biofilm and reducing probing pocket depths (PPD) [3]. Dental plaque and calculus are removed from tooth surfaces with the combined use of manual curettes/scalers and/or powered sonic or ultrasonic cleaning devices [4]. Non-surgical therapy leads to a reduction of tissue inflammation and subsequently to lower PPD and an improved clinical attachment level [5].

More and more evidence emerges that periodontal treatment improves not only oral health but also general health. The achieved benefits in oral health due to periodontal treatment were associated with improvement in endothelial function [6] and modulation of periodontitis-associated platelet activation [7].

However, intensive periodontal treatment also leads to local trauma and transient bacteraemia, resulting in acute, short-term systemic inflammation and endothelial dysfunction [6]. It is currently unknown if subgingival debridement also results in systemic platelet activation and therefore might bear a short-term risk for adverse thrombotic events in patients undergoing this treatment.

Therefore, it was the aim of this study to unravel if subgingival debridement causes systemic platelet activation to better estimate the short-term risk for thrombotic events in patients undergoing periodontal therapy.

## Material and methods

### Study design

Newly diagnosed patients with periodontitis (*n* = 26) presenting to the School of Dentistry, Medical University of Vienna, for periodontal treatment were recruited after initial periodontal screening (inclusion criteria: understanding of the study requirements, at least one interproximal site with a probing depth ≥ 5 mm and a loss of attachment at ≥ 2 interproximal sites ≥ 5 mm; exclusion criteria: all systemic diseases, pregnancy or breast feeding, antibiotics in the last 3 months and medication which affects platelets for at least 3 weeks before blood donation). They underwent a baseline periodontal examination and blood testing and full medical and dental histories were collected. Written informed consent was obtained from all study participants before study entry. The Ethics Committee of the Medical University of Vienna approved the study protocol (1656/2014), and the trial was conducted in accordance with the Declaration of Helsinki. The trial has been registered in the ISRCTN registry (#ISRCTN17035727), and the study uses the treatment group of a larger clinical trial that investigated short-term and long-term impact of subgingival debridement on platelet function and activation for analysis [7]. Patients donated blood before (time point PRE, 0 h) and immediately after intensive periodontal treatment (time point POST, 1–2 h) at the first treatment session for subgingival debridement.

### Periodontal examination and therapy

Clinical attachment level (CAL), comprising both periodontal probing depth and recession of the gingival margin relative to the cementoenamel junction at six sites per tooth, was examined at baseline with a CP 12 periodontal probe (Hu-Friedy, Frankfurt, Germany). The presence or absence of supragingival dental plaque and gingival bleeding on probing was also recorded. Intensive periodontal treatment was performed by subgingival debridement with curettes and sonic instruments (Sonicflex, KAVO, Biberach, Germany) generally in two to four treatment sessions after the initial examination with a time frame of 1 week or shorter between the sessions. Each session of subgingival debridement lasted for 1–2 h.

### Blood collection and platelet function tests

Venous blood was drawn by the antecubital vein with a 20 G needle and anticoagulated with 3.8% sodium citrate or ethylenediaminetetraacetic acid (EDTA). Blood count was determined with a Sysmex XP-300™ (Sysmex, Vienna, Austria) using EDTA blood. Platelet-rich plasma (PRP) was obtained by centrifugation of citrated blood at 125×*g* for 20 min.

All determinations were performed in duplicate. Platelet function tests were carried out to quantify platelet activation levels without any stimulus (referred to as basal) and to estimate their tendency to become activated by a physiologically relevant stimulus to test platelet reactivity. For measurement of basal platelet activation, phosphate-buffered saline (PBS) was applied instead of agonists. For assessment of platelet reactivity, platelet agonist adenosine diphosphate (ADP, Sigma Aldrich, Vienna, Austria) was incubated with platelets at different concentrations (final concentrations: 2.5, 5 and 50 μM) for 10 min.

Surface expression of P-selectin (CD62P) in unstimulated PRP (basal) represented the primary study outcome. Additional platelet activation markers as well as platelet reactivity in response to ADP stimulation were determined as secondary outcome parameters. Platelets were analysed by flow cytometry (Accuri C6, BD Biosciences, Franklin Lakes, NJ, USA) with analysis software (Accuri C6 software, BD Biosciences) as described previously [7].

### Statistical analyses

This study represents an exploratory analysis of data acquired in a recent randomised controlled trial [7] with an a priori determined sample size of 26 per group, and no additional sample size calculation was performed for the subgroup analysis. In the present study, only data of the treatment group (*n* = 26) were analysed. Based on the exploratory character of this study, no adjustment for multiplicity was performed, and results need to be interpreted accordingly. Platelet activation markers were analysed using mixed linear models with two fixed within-subjects factors (‘time point’ with the levels ‘PRE’ and ‘POST’, ‘ADP concentration’ with the four concentrations as levels) and patients as levels of a random factor. The Akaike information criterion was used to determine an adequate covariance structure. In case of a significant ‘time point’ × ‘ADP concentration’ interaction, contrasts were used to estimate the differences between time points separately at each ADP concentration. Otherwise, the non-significant interaction term was dropped from the model and the main effect of the time point was used as estimate applicable to all ADP concentrations. For the analysis of VASP phosphorylation, the within-subjects factor PGI (levels: basal, PGI) was used instead of the factor ADP concentration. Right-skewed data were log_10_ transformed prior to analysis. Statistical analysis was performed with IBM SPSS Statistics 25. Only two-sided tests were used. *P* values ≤ 0.05 were considered significant.

**Table 1:** Patient periodontal data: periodontal indices of the study participants; *PPD* periodontal probing depth, *CAL* clinical attachment level, *PESA* periodontal epithelial surface area, *PISA* periodontal inflamed surface area

	*n* = 26
Number of teeth	27.5 (26–29)
Plaque index, %	38.5 ± 19.5
Bleeding on probing, %	19.6 (9.4–29.7)
PPD ≥ 6 mm, number of sites per patient	17.5 (9–29)
PPD ≥ 6 mm, %	10.4 (6.0–18.5)
CAL ≥ 6 mm, number of sites per patient	27.5 (13–40)
CAL ≥ 4 mm, number of sites per patient	82 (55–104)
PESA, mm^2^	2060 ± 523
PISA, mm^2^	543.4 ± 425

#### Graphical representation

Baseline and post-treatment data of each group are presented as boxplots indicating median, 25th and 75th percentile, minimum and maximum. Platelet reactivity data (i.e. acquired in the presence of ADP or PGI) are shown as mean ± SEM.

## Results

We enrolled 26 patients with periodontitis with a median age of 45.5 (25–62) years, 38.5% were female and the medium body mass index was 23.6 (17.9–37.4) in this study. More than half of the patients (61.5%) were smokers. All patients showed normal blood counts. Mean bleeding on probing (BoP) of patients was at 19.6%. Mean CAL ≥ 6 mm was at 27.5 and CAL ≥ 4 mm at 82 numbers of sites per patient. Mean PPD ≥ 6 mm showed 17.5 numbers of sites per patient. The mean periodontal epithelial surface area (PESA) of the patients was 2060 ± 523 mm^2^, and the mean periodontal inflamed surface area (PISA) was 543 ± 425.5 mm^2^ [8]. The mean periodontal probing depth was 3.55 ± 0.6 mm. According to the 2018 classification of periodontal diseases and conditions [9], 84.6% of the included patients had periodontal disease of stage III and 15.4% of stage IV. For periodontitis progression, 42.3% were assigned a grade B and 57.7% a grade C (Table 1).

**Fig. 1:**
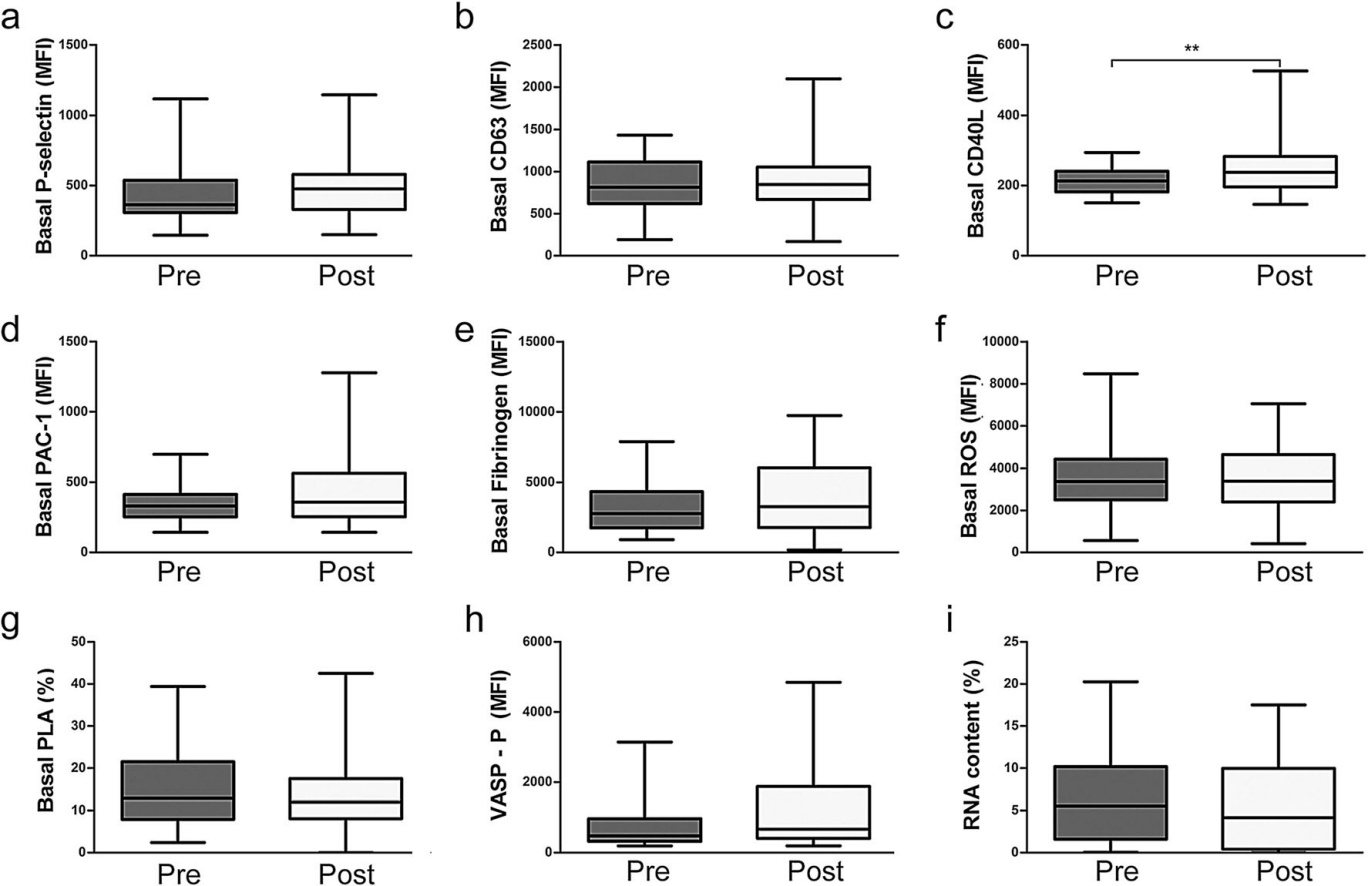
Effects of periodontal treatment on platelet function. Platelet activation state was determined by surface expression of P-selectin (**a**), surface expression of CD63 (**b**), surface expression of CD40L (**c**), binding of PAC-1 antibody, which represents GPIIb/IIIa activation (**d**), fibrinogen binding (**e**), intra-platelet reactive oxygen species (ROS) generation (**f**), platelet-leukocyte aggregate (PLA) formation (**g**), intra-platelet vasodilator-stimulated phosphoprotein (VASP) phosphorylation (**h**) and RNA positive platelets (**i**); dark grey, before periodontal treatment (PRE); light grey, immediately after periodontal treatment (POST), *n* = 26

No significant changes of platelet activation markers before and immediately after periodontal treatment could be observed (Fig. 1). Only CD40L surface expression was mildly but significantly elevated (Fig. 1c). To evaluate the systemic impact of this effect, we also determined soluble CD40L plasma levels in these patients but did not find significant differences between the two groups (data not shown). When we analysed the amount of reticulated platelets, we saw no difference before and after treatment (Fig. 1i). From these data, we conclude that no systemic platelet activation occurs in response to periodontal treatment.

**Fig. 2:**
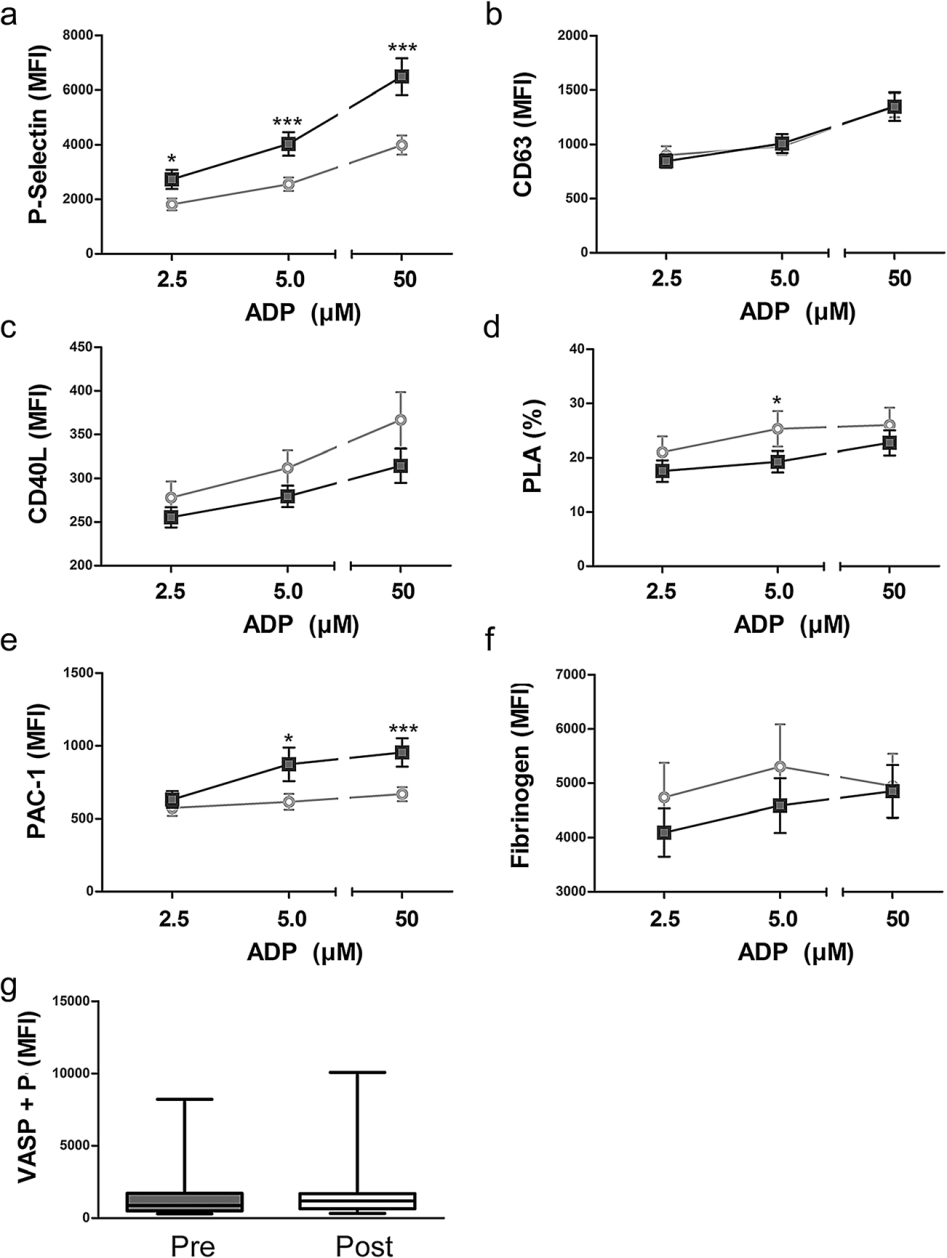
Effects of periodontal treatment on platelet response to different concentrations of ADP. Platelet response to ADP was measured after stimulation with 2.5, 5 and 50 μM ADP and responses monitored by surface expression of P-selectin (**a**), surface expression of CD63 (**b**), surface expression of CD40L (**c**), platelet-leukocyte aggregate (PLA) formation (**d**), binding of PAC-1 antibody, which represents GPIIb/IIIa activation (**e**), fibrinogen binding (**f**) and intra-platelet vasodilator-stimulated phosphoprotein (VASP) phosphorylation (**g**); dark grey, before periodontal treatment (PRE); light grey, immediately after periodontal treatment (POST), *n* = 26

We then investigated if periodontal treatment affects platelet reactivity in response to platelet agonist ADP. As depicted in Fig. 2, platelet activation in response to different concentrations of ADP did not show significantly enhanced platelet responses. On the contrary, in response to ADP, P-selectin surface expression of platelets was significantly reduced (Fig. 2a) and we further observed a reduction in integrin αIIbβ3 activation at moderate and high concentrations of ADP (Fig. 2e). This implies that even if platelets do become activated locally during periodontal treatment, no systemic platelet activation occurred.

## Discussion

In this study, we analysed systemic platelet activation in response to periodontal treatment by utilising a number of different platelet activation markers, including CD62P, CD63 and CD40L. CD62P is part of the platelet α-granule membrane, which fuses with the platelet membrane upon exocytosis, while CD63 represents a marker for dense granules and lysosomes. Also CD40L translocates to the platelet surface upon activation [10]. Therefore, these markers were investigated to determine platelet degranulation. However, in our experimental setting, we did not observe changes in CP62P and CD63 surface expression in response to subgingival debridement. CD40L was the only marker that mildly but significantly increased, while plasma levels of soluble CD40L remained unchanged. Platelets represent the main source of soluble CD40L, and elevated plasma levels of soluble CD40L are found in periodontitis patients [11]. Platelet CD40L is known to induce inflammatory responses in the endothelium [12], to initiate the formation of ROS and inhibition of nitric oxide production [13] and to foster the release of chemokines and the expression of several adhesion receptors as well as tissue factors [14]. Further, it plays a role in the progression of plaque formation, macrophage infiltration into the plaque and T cell homeostasis in the blood and spleen [15]. Since CD40L surface expression was only marginally increased and soluble CD40L remained unaltered, we conclude that subgingival debridement is unlikely to enhance CD40L-related cardiovascular complications.

We then determined platelet fibrinogen binding and activation of the fibrin receptor GPIIb/IIIa. GPIIb/IIIa represents the most abundant platelet glycoprotein and is central for platelet aggregation via fibrinogen bridging. GPIIb/IIIa becomes activated via inside-out signalling, leading to a conformational change that uncovers an arginine-glycine-aspartic acid (RGD) sequence, which allows for binding of fibrinogen, vWF, fibronectin and vitronectin [16]. As GPIIb/IIIa activation as well as fibrinogen binding remained unaltered in patients undergoing periodontal therapy, we conclude that inside-out signalling is not affected by this treatment. There was also no difference in platelet ROS formation, which further indicates that systemic platelet activation remained unaltered during subgingival debridement. There was no increase in platelet interaction with other blood cells, measured by PLA formation, an important marker of inflammatory platelet-leukocyte interactions [17]. Also VASP phosphorylation that represents an important marker of platelet inhibition, which is influenced, e.g. by smoking [18], remained unaltered.

Platelet RNA content represents a surrogate marker for newly formed immature platelets which are also called reticulated platelets due to the presence of residual cytosolic mRNA [19]. Reticulated platelets are known to be more reactive than older platelets and promote platelet aggregate formation [20] and are indicative for a higher platelet turnover. However, no impact of subgingival debridement on platelet RNA content could be observed in our study. Tonetti et al. [6] and Eickholz et al. [21] found marked systemic effects after subgingival debridement. Although the severity of our study population is comparable, as Tonetti et al. reported a mean number of 84 ± 26 periodontal lesions per patient and our patients show a mean number of 82 ± 33 periodontal lesions per patient, Eickholz et al. reported a mean periodontal probing depth of 3.5 ± 0.7 mm and in our treatment group we found a mean probing depth of 3.55 ± 0.6 mm; the difference in the outcomes might be attributed to the timing of the subgingival debridement. In our study, subgingival debridement was completed in 2–4 sessions with several days in between. Blood was taken after the debridement of only 1–2 quadrants whereas Eickholz et al. performed subgingival debridement in 1–2 visits at two consecutive days and Tonetti et al. made a full mouth intensive subgingival debridement. The extent of the subgingival debridement might be an explanation for the different findings.

Our result suggests that even if platelets become locally activated by the periodontal treatment, systemically no increase in platelet activation can be detected. We previously observed a similar phenomenon in healthy volunteers that were challenged with an endotoxin stimulus [22]. While platelets did locally respond to the toxin, systemically no increase in platelet activation occurred [22], suggesting that activated platelets may get scavenged and are therefore no longer detected in the circulation.

Of note, all patients were followed up for a period of 3 months and platelet activation markers were then investigated again and compared with a control cohort. We found that periodontal treatment was able to limit platelet activation in patients with periodontitis [7], indicating beneficial systemic effects of periodontal therapy.

Our investigation has certain limitations. Firstly, the number of individuals included in this analysis is relatively small and therefore the findings should be interpreted with caution. Further, we only investigated a single—very early—time point. It is possible that platelet activation and/or reactivity increase at later time points, where also endothelial cell and leukocyte activation were detected. It is known that intensive periodontal therapy induces a moderate acute systemic inflammatory response associated with endothelial cell activation and mildly increased plasma cytokine levels [6, 21, 23, 24]. However, this implies that platelets get only indirectly activated by other cells and not due to the treatment as such. Additionally, activated platelets might get scavenged and therefore might not be longer detectable in the circulation as observed in previous studies in response to inflammatory stimuli [22]. In this study, we did not include a control group, since blood was taken immediately before and immediately after gingival debridement. So both blood samples were taken within less than 2 h, and the first time point served as a baseline measurement for the second time point. It is known that platelet activation is enhanced in patients with periodontitis [11, 25, 26] but no further enhancement could be observed upon subgingival debridement.

From our data, we conclude that subgingival debridement does not result in elevated platelet activation and therefore does not seem to increase the immediate platelet-related adverse events.
